# Thalamic Atrophy Predicts 5-Year Disability Progression in Multiple Sclerosis

**DOI:** 10.3389/fneur.2020.00606

**Published:** 2020-07-15

**Authors:** Katariina Hänninen, Matias Viitala, Teemu Paavilainen, Jari O. Karhu, Juha Rinne, Juha Koikkalainen, Jyrki Lötjönen, Merja Soilu-Hänninen

**Affiliations:** ^1^Neurocenter, Turku University Hospital, University of Turku, Turku, Finland; ^2^Department of Mathematics and Statistics, University of Turku, Turku, Finland; ^3^StellarQ Ltd., Turku, Finland; ^4^Medical Imaging Centre of Southwest Finland, Turku, Finland; ^5^Turku PET Centre, University of Turku, Turku, Finland; ^6^Combinostics Ltd., Tampere, Finland

**Keywords:** multiple sclerosis, thalamus, brain atrophy, disability, MRI, EDSS

## Abstract

**Purpose:** Thalamus is among the first brain regions to become atrophic in multiple sclerosis (MS). We studied whether thalamic atrophy predicts disability progression at 5 years in a cohort of Finnish MS patients.

**Methods:** Global and regional brain volumes were measured from 24 newly diagnosed relapsing MS (RMS) patients 6 months after initiation of therapy and from 36 secondary progressive MS (SPMS) patients. The patients were divided into groups based on baseline whole brain parenchymal (BP) and thalamic atrophy. Standard scores (*z* scores) were computed by comparing individual brain volumes with healthy controls. A *z* score cutoff of −1.96 was applied to separate atrophic from normal brain volumes. The Expanded Disability Status Scale (EDSS), brain magnetic resonance imaging (MRI) findings, and relapses were assessed at baseline and at 2 years and EDSS progression at 5 years.

**Results:** Baseline thalamus volume predicted disability in 5 years in a logistic regression model (*p* = 0.031). At 5 years, EDSS was same or better in 12 of 18 patients with no brain atrophy at baseline but only in 5 of 18 patients with isolated thalamic atrophy [odds ratio (OR) (95% CI) = 5.2 (1.25, 21.57)]. The patients with isolated thalamic atrophy had more escalations of disease-modifying therapies during follow-up.

**Conclusion:** Patients with thalamic atrophy at baseline were at a higher risk for 5-year EDSS increase than patients with no identified brain atrophy. Brain volume measurement at a single time point could help predict disability progression in MS and complement clinical and routine MRI evaluation in therapeutic decision-making.

## Introduction

Multiple sclerosis (MS) is the most common chronic, progressive neurological disease among young adults. Inflammation, demyelination, and axonal loss in the central nervous system occur from early on in the disease course. Neurodegenerative processes act as one of the major contributors to the long-term disability accumulation in MS (1). The emergence of effective disease-modifying treatments (DMTs) has emphasized the importance to initiate treatment as early as possible. Choosing the correct treatment for an MS patient requires careful consideration of disease activity, treatment efficacy, and side-effect profile (2). In clinical practice, patients are typically selected for the most intensive therapies on the basis of clinical and radiological features predictive of a poor outcome, but more sophisticated predictive models to capture prognosis and treatment response are needed (3).

Magnetic resonance imaging (MRI)-derived brain volume measures are among the most extensively investigated biomarkers for quantifying neuroaxonal injury and neurodegeneration in MS (4). Several studies have shown that brain atrophy is a relevant marker of disease progression (5–7), and especially the deep gray matter areas, thalamus and putamen, have risen as promising biomarkers in MS (8, 9).

The aim of our work is to assess the usefulness of single time point measurement of global and regional brain volumes as a predictive tool to capture risk of MS progression. We previously showed that MS patients with isolated thalamic atrophy, detected at a single time point measurement, were at a higher risk for EDSS increase and not reaching NEDA-3 at 2 years, than patients with no identified brain atrophy (10). In the current study, we investigated the disability progression of the same clinical cohort of MS patients at 5 years after baseline. We performed a logistic regression analysis to study whether EDSS increase at 5 years could be explained by any of the baseline gray matter volumes.

## Patients and Methods

### Subjects

At study onset, 24 patients with newly diagnosed RMS on first-line immunomodulatory treatment initiated 6 months before study baseline and 36 SPMS patients were included. Inclusion criteria for the RMS patients were (1) RMS fulfilling McDonald 2010 criteria, (2) EDSS 0–3.5, and (3) either glatiramer acetate of interferon-beta treatment initiated within 12 months as the first DMT. In the SPMS group, the inclusion criteria were (1) patients who had entered secondary progressive stage of MS, as defined by the treating neurologist and (2) Expanded Disability Status Scale (EDSS) 4–6.5. All the participants were patients at the outpatient clinic of Turku University Hospital. Exclusion criteria were the following: contraindications to MRI, malignancy, pregnancy or planning of a pregnancy, and failure to obtain informed consent from the patient. The 2-year follow-up data of the same patients have been previously published by our study group (10).

### Clinical Evaluation

The following background data were collected: sex, age, date of MS diagnosis, neurological status findings, first symptoms, serum concentration of 25-hydroxyvitamin D [25-OH(D)], socioeconomic status, EDSS, use of disease-modifying therapies, and data on relapses from the date of first symptoms until the end of the study. All background and follow-up data were collected from the Turku University Hospital electronic patient files using structured StellarQ MS registry (www.stellarq.com). The first patient enrolled in March 2014 and last patient in January 2015.

The 5-year analysis included information on relapses, annual EDSS, MRI findings at the study baseline, immunomodulatory drug usage, and possible changes in treatment during the follow-up. EDSS increase was defined by a one-point increase in EDSS in patients with baseline EDSS <5.5 (a 1.5-point increase for EDSS = 0) or an increase of 0.5 for patients with an EDSS of 5.5 or greater (11, 12).

### Ethics Committee Approval

This study has been approved by the Ethics committee of Turku University Hospital and University of Turku at 21.1.2014. A written informed consent was obtained from all patients participating in the study.

### MRI Acquisition and Analysis

MRIs were obtained from the 24 RMS patients 6 months after initiating DMT and from the 36 SPMS patients at study baseline. All of the images were analyzed by the same neuroradiologist (JOK) with long experience in MS image analyses. The patients had to be clinically stable and have no corticosteroid administration within 30 days before the MRI. All of the brain MRIs were acquired at the same radiological facility using the same scanner and same acquisition protocol settings for all of the scans. Timing of the MRI scans was based on a routine clinical practice in Finland to rescan all MS patients at 6 months after initiation of DMTs (13).

The magnetic resonance images were obtained using a Siemens Skyra 3.0 T scanner. The MRI analysis methods have been described in our previous paper (10). Briefly, the MRI examinations included the acquisitions of high-resolution pre- and post-contrast 3D T1-weighted sequence and a 3D fluid attenuated inversion recovery (FLAIR) sequence. T1-weighted images were acquired using a 3D magnetization-prepared rapid gradient-echo sequence (MPRAGE) in sagittal planes [repetition time (TR) = 2,300 ms; echo time (TE) = 2.29 ms; inversion time (TI) = 900 ms; flip angle = 8°]. FLAIR images were acquired in sagittal planes (TR = 5,000 ms; TE = 396 ms; TI = 1,800 ms; flip angle = 120°). Resolution of the T1 images was 0.94 × 0.90–1.00 × 0.90–1.00 mm and of the FLAIR images was 1.00 × 0.94–1.00 × 0.94–1.0 mm and slice thickness was 3 mm.

#### Brain Volume Measurement

Total brain parenchyma (BP) volume, lesion volumes, total white and gray matter volumes, cerebellum, and deep gray matter volumes were determined from the 3D T1 MRI images, which were obtained prior to gadolinium administration, as described previously (10, 14, 15). Volumes normalized for age, sex, and head size were used in the statistical analyses. The cNeuro method (Combinostics Ltd, Tampere, Finland) is a CE marked tool for brain atrophy measurement and is in clinical use at the Turku University Hospital. The segmentation method described by Koikkalainen et al. (15) and Wang et al. (16) was used to compute volumes of white-matter lesions from 3D FLAIR images. An example of brain volume measurement by cNeuro is shown in Supplemental Figure 1.

### Standard Score (*z* score) Calculation and Grouping of MS Patients

Standard scores (*z* scores) were defined by comparing individual brain volumes with corresponding volumes from the Open Access Series of Imaging Studies (OASIS) (17) cohort of 295 healthy controls acquired using Siemens 3 T scanners, which showed relatively consistent results: the 95% confidence interval of the average thalamus volume for each scanner contains the average thalamus volume computed for all eight scanners. A *z*-score cutoff of −1.96 was applied to separate pathologically atrophic from normal brain volumes for thalamus and whole brain parenchymal (BP) volume (accepting a 2.5% error probability).

Based on *z* scores, the patients were divided into groups with no brain atrophy, isolated thalamic atrophy, both thalamic and whole BP atrophy, and whole BP atrophy without thalamic atrophy as described earlier by Raji et al. and us (10, 18). The group that had no thalamic or whole BP atrophy was named group 1, the group with thalamic atrophy but no whole BP atrophy was named group 2, and the group with both thalamic and whole BP was named group 3. The grouping was performed as such because we aimed to study whether the difference in disability progression we found between groups 1 and 2 in our previous study with the same patients (10) was still detectable at 5 years after baseline. There was one patient who had whole BP atrophy without thalamic atrophy and could not be classified into these three groups.

### Statistical Analysis

The baseline group comparison *p*-values for categorical variables were calculated using Fisher's exact test where conditional probabilities are obtained from hypergeometrical distribution (19). Group comparison *p*-values for continuous variables were calculated using non-parametric Wilcoxon rank-sum test testing location shift difference between two samples (20). *p*-values in Tables 1, 2 were adjusted using the Benjamini and Hoghberg method (1995), where false discovery rate (FDR) is controlled penalizing smaller *p*-values more than higher *p*-values (21).

**Table 1:** Baseline demographic, clinical, and MRI characteristics of the patients with and without thalamus atrophy.

**Variable**	**Group1**	**Group 2**		**Group 3**	
			*p*-value^*^		*p*-value^*^
*N*	19	18	22		
Age, years (mean, SD)	42.8 (10.04)	40.5 (10.39)	0.860	53.2 (7.97)	**0.002**
RMS/SPMS	14/5	10/8	0.903	2/20	**0.001**
Female/male	17/2	13/5	0.696	10/12	**0.006**
Disease duration, years (mean, SD)	4.9 (6.07)	9.9 (11.54)	0.903	20.5 (10.63)	**<0.001**
EDSS (median, range)	2.0 (0.0–6.5)	1.8 (0.0–6.5)	0.970	4.0 (2.0–6.0)	**0.040**
Education, years (mean, SD)	13.0 (2.62)	11.7 (2.35)	0.403	12.7 (2.97)	0.719
SDMT (mean, SD)	48.5 (10.87)	45.1 (12.56)	0.818	33.1 (10.05)	**<0.001**
BMI kg/m^2^ (mean, SD)	27.3 (5.57)	24.9 (3.86)	0.696	25.5 (4.72)	0.602
Smoking, yes/no	7/12	10/8	0.720	7/15	0.822
25(OH)D, nmol/L (mean, SD)	118.9 (53.58)	111.5 (48.63)	0.903	112.8 (60.3)	0.616
Number of relapses (mean, SD)	0.4 (0.69)	0.5 (0.92)	0.970	0.1 (0.47)	0.120
Total BP vol. (ml, SD)	1505.3 (93.33)	1476.7 (111.44)	0.903	1354.0 (101.58)	**<0.001**
Total WM vol. (ml, SD)	694.2 (43.38)	672.8 (48.78)	0.709	628.5 (47.30)	**<0.001**
Total GM vol. (ml, SD)	810.8 (59.14)	803.9 (69.55)	0.970	725.7 (65.83)	**<0.001**
Cortical GM vol. (ml, SD)	474.3 (18.24)	477.5 (19.52)	0.970	462.3 (23.48)	0.161
WM lesion vol. (ml, SD)	11.4 (5.53)	14.7 (6.75)	0.322	26.6 (16.99)	**<0.001**
Putamen vol. (ml, SD)	3.9 (0.40)	3.8 (0.35)	0.822	3.4 (0.53)	**<0.001**
Hippocampus vol. (ml, SD)	3.4 (0.24)	3.4 (0.33)	0.818	3.0 (0.32)	**<0.001**
Thalamus vol. (ml, SD)	6.6 (0.43)	5.7 (0.41)	**<0.001**	5.3 (0.78)	**<0.001**
Nucleus caudatus vol. (ml, SD)	2.8 (0.24)	2.6 (0.32)	0.194	2.3 (0.32)	**<0.001**
Globus pallidus vol. (ml, SD)	1.3 (0.14)	1.2 (0.12)	0.322	1.1 (0.12)	**<0.001**
Cerebellum GM vol. (ml, SD)	47.9 (4.89)	46.2 (4.59)	0.903	8.9 (1.02)	**<0.001**
Cerebellum WM vo. (ml, SD).	11.1 (1.24)	10.5 (0.97)	0.322	47.4 (6.45)	0.883
Gd + lesions (*N*, %)	0/19 (0.0)	5/18 (27.8)	0.194	1/22 (4.5)	1.000

Group 1: no brain atrophy at baseline; group 2: isolated thalamic atrophy at baseline; group 3: whole brain and thalamic atrophy at baseline.

* *p-values are calculated against group 1*.

**Table 2:** Logistic regression for prediction of disability in 5 years by baseline thalamic volume.

**Variable**	**Estimate**	**Std. erro**	***p*-value**	**OR (95% CI)**
Intercept		6.46	3.034	0.033
Age	−0.04	0.027	0.196	0.95 (0.91, 1.02)
Thalamus vol.	−0.86	0.398	**0.031**	0.42 (0.18, 0.88)

^*^57 patients included in logistic regression model.

The prevalence, sensitivity, specificity, positive predictive value, and negative predictive value derivations were calculated for EDSS change in groups 1–3 as described by Akobeng et al. (22). Odds ratios are maximum likelihood estimates derived using the log-likelihood of the odds (23).

Reaching EDSS increase in 5 years was modeled with the whole study population where all baseline MRI covariates and 5-year EDSS could be assessed (57/60 patients). One patient deceased, and two lost to follow-up. Dependent variable reaching EDSS increase in 5 years is represented as binary (0/1) variable. All MRI variables in Table 1 were taken into account to explain EDSS increase, except for total WM volume and cerebellum WM volume due to the fact that gray matter atrophy has shown to have a greater clinical relevance in MS (1, 24). Age at baseline was added to adjust baseline characteristics of modeled population. Logistic regression model was fitted using generalized linear model with logit link function to explain EDSS increase (25). Authors recognize the limits of small population in this study and possible underlying multicollinearity between MRI variables. Four most important covariates (thalamus vol., putamen vol., WM lesion vol., and globus pallidus vol.) were identified based on univariate analysis. Underlying multicollinearity was checked, and usage of selected variables was confirmed using variance inflation factors (VIFs). The best model was selected using manual forward selection where simple model is the starting point and terms were added based on deviance analysis until the model does not significantly improve. In addition, automatic forward and backward Akaike Information Criteria (AIC)-based stepwise algorithms support the model choice. The final model and its estimates are represented in Table 2. Diagnostic checks were made to detect possible influential outliers. A few outliers were detected, but based on studentized residuals and Cook's distance, they were not influential enough to justify refitting model without those observations (26, 27).

## Results

### Disability Progression in Groups of Patients Formed Based on Identification of Isolated Thalamus Atrophy

The baseline demographic and clinical characteristics and MRI results of the patients are shown in Table 1. The group that had no thalamic and no BP atrophy was named group 1 (*n* = 19). The group with thalamic atrophy without whole BP atrophy was named group 2 (*n* = 18). The group with both thalamic and whole brain atrophy was named group 3 (*n* = 22). There was no significant difference in the demographic and clinical characteristics between groups 1 and 2, but in group 3, the patients were older and had a more advanced disease at baseline compared to group 1.

### Logistic Regression Analysis of Baseline Brain Volumes Predicting Disability in 5 Years

Reaching EDSS increase in 5 years was modeled with the whole study population where 5-year EDSS could be assessed. The final model and its estimates are presented in Table 2. Interpretation of the results of the logistic regression model: baseline thalamus volume was significant (*p* = 0.031). Every unit decrease in baseline thalamus volume increased the odds to reach disability progression over 5 years to 2.4-fold.

The prevalence, sensitivity, specificity, and positive and negative predictive values for EDSS change at 5 years in groups 1–3 are shown in Table 3. At 5 years, EDSS was the same or better in 12 of the 18 patients in group 1, while in group 2, EDSS was the same or better only in 5 of the 18 patients. In group 3, EDSS was the same or better in 7 of the 22 patients.

**Table 3:** The predictive value of baseline brain atrophy measures to EDSS change at 5 years.

	**Group 1 vs**.	**Group 1 vs**.	**Group 1 vs**.
	**Group 2**	**Group 3**	**Group 2 + 3**
Prevalence	53%	52%	59%
Sensitivity	68%	71%	82%
Specificity	71%	63%	50%
**Positive predictive**			
Value	72%	68%	70%
**Negative predictive**			
Value	67%	67%	67%
Odds ratio (OR)			
(95% CI)	5.2 (1.25, 21.57)	4.3 (1.14, 16.18)	4.7 (1.42, 15.35)

*All the comparisons were made using patients without brain atrophy at the baseline (group 1) as the reference group. Group 2 = isolated thalamic atrophy; group 3 = whole brain parenchyma atrophy. Group 1: EDSS same/better (n) = 12, EDSS worse (n) = 6, one patient excluded because of missing control value. Group 2: EDSS same/better (n) = 5, EDSS worse (n) = 13. Group 3: EDSS same/better (n) = 7, EDSS worse (n) = 15*.

### DMT Changes in Patients With and Without Thalamus Atrophy

**Figure 1:**
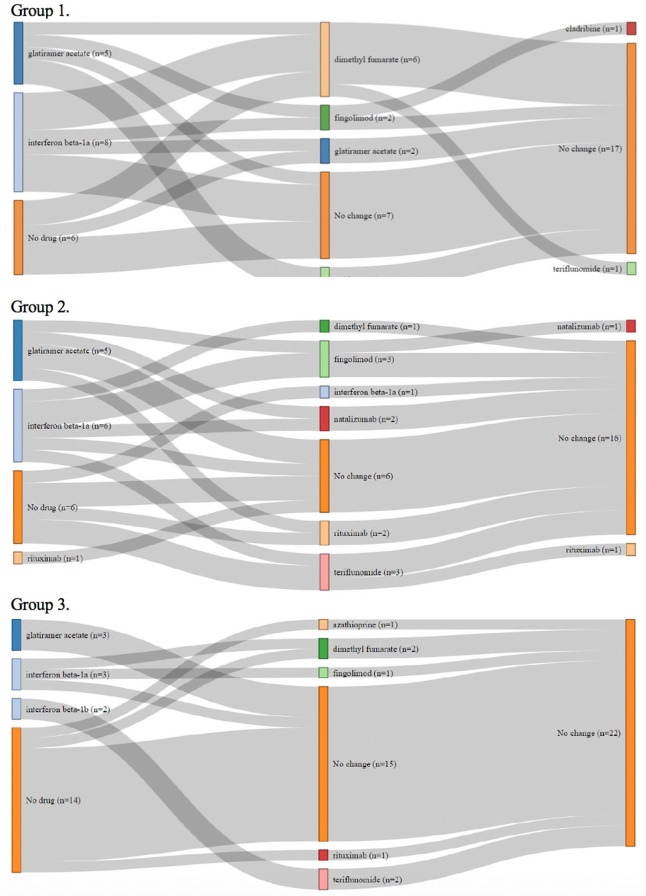
Changes of disease-modifying therapies during the study in the patients without brain atrophy at the study baseline (group 1), in patients with isolated thalamic atrophy (group 2), and in patients with whole brain parenchyma atrophy (group 3).

Changes in DMT during the 5-year follow-up are presented in Figure 1. A total of 13 patients in group 1 had either interferon or glatiramer acetate therapy at the start of the study, and 3 of them (21%) escalated therapy into fingolimod and cladribine during the study. In group 2, a total of 11 patients had interferon or glatiramer acetate therapy at baseline, and 7 of them (64%) escalated therapy into natalizumab, fingolimod, or rituximab during the study. In group 2, more patients went through escalation of therapy than in group 1 (*p* = 0.017), which indicates that they had a more active disease. One SPMS patient in group 2 used rituximab throughout the study. In group 3, most of the patients (14/22) were not receiving DMTs, five patients were on interferon beta or glatiramer acetate therapy, and one of them escalated therapy into fingolimod.

## Discussion

In this paper, using conditional logistic regression, we found that every unit decrease in baseline thalamus volume increased the odds to reach disability progression over 5 years to 2.4-fold. We further wished to delineate the role of thalamus as a predictor of disability progression by grouping of the patients into those that had only developed thalamus atrophy, but not yet more widespread brain atrophy, when compared to healthy controls. The results suggested that detection of isolated thalamus atrophy predicted EDSS increase more significantly than the measure of thalamus volume as such. The patients that had already developed whole brain parenchymal atrophy (group 3) were also at increased risk for 5-year disability progression compared to those with no brain atrophy. However, these patients were older and had a more advanced disease at the study baseline, whereas the patients with isolated thalamic atrophy where clinically indistinguishable from the patients with no brain atrophy at baseline. The odds ratio for disability progression was higher in the group of patients with isolated thalamic atrophy than in the patients with whole brain parenchymal atrophy. This supports the view that thalamic atrophy might be a more sensitive tool than whole brain atrophy in predicting future disability in MS.

Our results are in line with previous studies showing that thalamic atrophy correlates with disability in MS. In a prospective, 8-year follow-up study with 73 relapse-onset MS patients, thalamic atrophy was shown to correlate with long-term accumulation of disability (28). In a large, retrospective multicenter study, smaller deep gray matter (DGM) volume at baseline was associated with increased risk of shorter time to EDSS progression over an average follow-up of 2.41 years (29). In a *post-hoc* analysis of two randomized placebo-controlled phase III trials, Gaetano et al. showed that lower thalamic volume at baseline was associated with disability progression, regardless of treatment (30). In their editorial commenting the study by Gaetano et al., Schoonheim and Ciccarelli suggested including thalamic atrophy as a trial endpoint in clinical trials with disease-modifying treatments in MS (31).

Many studies regarding brain atrophy measures in MS have been in a setting of randomized clinical trials. In a study where CIS patients were treated with intramuscular interferon beta, thalamic atrophy was related to conversion of clinically isolated syndrome (CIS) to clinically definite MS (32). In a 2-year, double-blind, placebo-controlled Avonex-Steroid-Azathioprine study, thalamic atrophy was related to disability progression in early relapse-onset MS (33). However, the use of DMTs in real world is often more complex. The MS patients participating in our study were treated at the outpatient clinic of the Turku University Hospital according to the Finnish MS treatment guidelines (13) and therefore reflect the normal MS clinical practice in our country. During our study, the patients with isolated thalamic atrophy went through more DMT escalations than those without thalamic atrophy, suggesting that they had a more severe disease, even though the groups were clinically indistinguishable at baseline.

In clinical practice, patients are typically selected for early intensive therapies on the basis of clinical and radiological features predictive of a poor outcome. Recent studies have suggested that the contemporary surveillance strategies and escalation protocols may not be sufficient to identify all the patients with unfavorable outcomes (34, 35). Our results support adding brain volume measures to previously described treatment failure predictors to increase sensitivity to early identification of non-responder patients to platform DMTs.

Although having the potential as a future MS biomarker, brain atrophy measurement is not yet recommended for use in MS imaging guidelines (36). There are several reasons for this, including the confounding factors involved in brain volume measurement and lack of established guidelines on the implementation of the imaging and image analysis. To avoid error caused by technical issues, such as impact of scanner type and patient positioning, the same scanner and same imaging protocol should be used when measuring change of volume in time. This is seldom achievable in clinical setting. A single time point atrophy measure would be more feasible to implement in clinical practice and would save the years of follow-up when brain atrophy can already progress and the therapeutic window for its prevention close. It is obvious that a single time point prognostic volumetric assessment would not remove the need for developing means to assess the rate of brain atrophy in time when evaluating therapeutic response to MS DMTs.

The major limitation of our study is the small number of patients especially in the subgroup analyses. The different treatments between patient groups and changes in treatment during the study can be regarded as a limitation because the changes may influence the risk of disability progression (32). The strength of this study is that the confounding factors, which could have had an effect on brain volumes, such as age, sex, smoking and vitamin D status, BMI, clinical disease activity, disease duration and disability, did not differ between the patients with and without isolated thalamic atrophy. In addition, all of the brain MRIs were acquired with the same Siemens 3 T MRI scanner and using the same imaging protocol. The *z* scores were obtained from the OASIS cohort of 295 healthy controls. Although only Siemens 3 T scanners showing relatively consistent results were used as the controls, it would have been ideal to use the same scanner as for the MS patients. In other words, the use of different scanners may have impacted, for example, on the number of the SPMS cases in group 1 because, especially for the thalamus, all the SPMS cases were relatively close to the *z*-score cutoff. We should neither oversee other reasons that may move a single patient across the cutoff, e.g., inaccuracies in automated volume measurements or in normalization of age, sex, and head size.

Further long-term follow-up studies, with larger patient cohorts are needed to establish whether a single time point measure of deep gray matter atrophy could be useful as a predictor of disability progression in MS.

## Conclusion

Measurement of thalamic atrophy could help predict future disability progression and complement clinical and routine MRI evaluation in clinical decision-making. For brain atrophy measures to become integrated into clinical practice, unified guidelines on how to implement the measurements, which segmentation tools to use, and which brain regions to focus on are needed.

## Data Availability Statement

The datasets generated for this study are available on request to the corresponding author.

## Ethics Statement

The studies involving human participants were reviewed and approved by The study was approved by the Ethics Committee of Turku University Hospital and University of Turku at 21.1.2014. The study was performed in accordance with the ethical standards laid down in the 1964 Declaration of Helsinki and its later amendments. The patients/participants provided their written informed consent to participate in this study.

## Author Contributions

KH: study design, analysis, drafting and revision of manuscript and corresponding author. MV: data analysis and revision of manuscript. TP and JOK: MRI image analyses and revision of manuscript. JR: study design and revision of manuscript. JK and JL: study design, MRI analyses and revision of manuscript. MS-H: study design, data analyses and revision of manuscript. All authors: contributed to the article and approved the submitted version.

## Conflict of Interest

MS-H has received honoraria for advisory boards, lectures, or for working as an investigator for clinical trials from Biogen, Bayer, Eisai, Orion, Novartis, Roche, Sanofi-Genzyme, Teva, and UCB. MV has been an employee at StellarQ since October 2018. JL and JK are shareholders and employees at Combinostics. TP has received a lecture fee from Roche. JR serves as a consultant neurologist for Clinical Research Services Turku (CRST). The remaining authors declare that this research was conducted in the absence of any commercial or financial relationships that could be construed as a potential conflict of interest.

## Supplementary Material

Supplemental Figure 1 Example of brain segmentation by cNeuro: Upper left panel shows a T1 image of one of the study patients and upper right its segmentation for the different brain region volume measurement. Lower left panels shown a FLAIR image of the same patients and lower right panel white matter MS lesion detection for the automated lesion volume measurement by cNeuro.
