# Supplementary figures and images for: Thalamic Atrophy Predicts 5-Year Disability Progression in Multiple Sclerosis
Source: Front Neurol. 2020 Jul 15;11:606. doi: 10.3389/fneur.2020.00606 (PMC7373757; doi:10.3389/fneur.2020.00606)

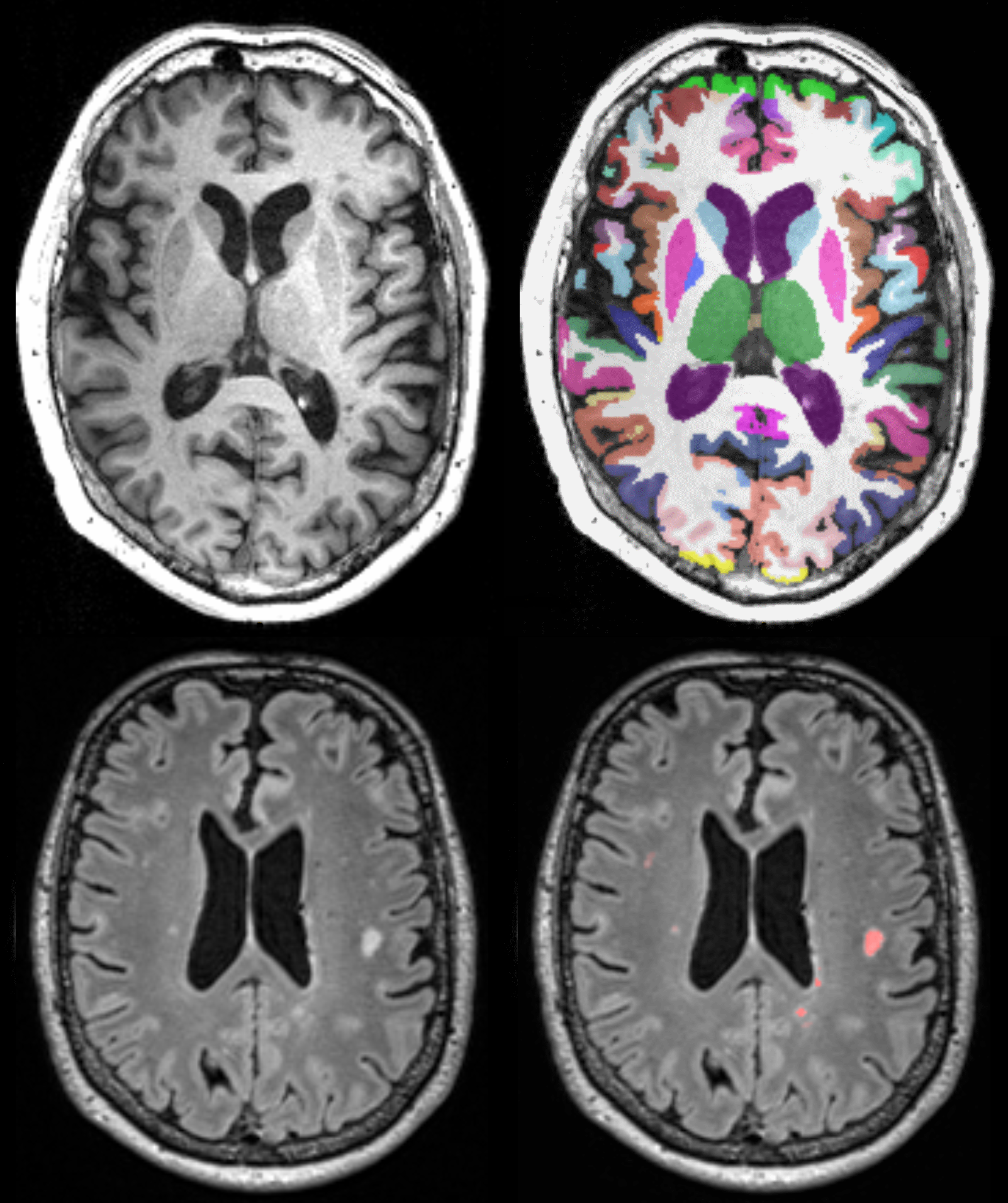

Supplement: Supplemental Figure 1 — Example of brain segmentation by cNeuro: Upper left panel shows a T1 image of one of the study patients and upper right its segmentation for the different brain region volume measurement. Lower left panels shown a FLAIR image of the same patients and lower right panel white matter MS lesion detection for the automated lesion volume measurement by cNeuro. [file Image_1.TIFF]
